# Impact of fat intake on [^18^F]AlF-NOTA-FAPI-04 uptake in normal abdominal organs

**DOI:** 10.3389/fmed.2024.1464779

**Published:** 2024-11-07

**Authors:** Jiashun Dai, Wanjing Zhou, Huaping Liu, Chengzhi Jiang, Hui Ye

**Affiliations:** ^1^Department of PET-CT Center, The Affiliated Cancer Hospital of Xiangya School of Medicine, Central South University/Hunan Cancer Hospital, Changsha, China; ^2^Department of Radiology, The Affiliated Cancer Hospital of Xiangya School of Medicine, Central South University/Hunan Cancer Hospital, Changsha, China

**Keywords:** milk, [^18^F]AlF-NOTA-FAPI-04, positron emission tomography, gallbladder, SUV_mean_

## Abstract

**Purpose:**

[^18^F]AlF-NOTA-FAPI-04 demonstrates significant physiological uptake in the gallbladder and biliary tract system, representing a limitation of this positron emission tomography (PET) tracer. The aim of this study was to evaluate the impact of milk consumed prior to a PET/CT scan on [^18^F]AlF-NOTA-FAPI-04 uptake in normal abdominal organs.

**Materials and methods:**

A total of 86 patients who underwent [^18^F]AlF-NOTA-FAPI-04 PET/CT imaging took part in this single-center retrospective clinical study at the Hunan Cancer Hospital between December 2020 and August 2021. Patients were divided into two groups according to their pre-PET scan diet: treated group, who consumed 250 mL of milk 10 ± 5 min after the tracer injection, while the control group was permitted no food intake subsequent to the radiotracer administration. The mean standardized uptake value (SUV_mean_) of gallbladder, liver, small intestine and pancreas were measured in ^18^F-FAPI and ^18^F-FDG PET/CT.

**Results:**

There was a statistically significant difference in the ^18^F-FAPI uptake in the gallbladder between the treated group and the control group (*p* < 0.001). The average SUV_mean_ in the treated group was 2.19 ± 2.01, which was significantly lower than the average SUV_mean_ of 10.04 ± 9.66 in the control group. In the subgroup analysis of patients who underwent paired [^18^F]FDG and [^18^F]FAPI PET/CT scans, the ^18^F-FAPI uptake of liver and small intestine was significantly lower than the ^18^F-FDG uptake in both the treated group and the control group (*p* < 0.001).

**Conclusion:**

This study suggests that milk consumption decreases physiological ^18^F-FAPI uptake in the gallbladder, potentially enhancing the diagnostic accuracy for gallbladder cancer.

## Introduction

Fibroblast activation protein (FAP), highly expressed in cancer-associated fibroblasts, is a type II transmembrane glycoprotein enzyme with peptidase activity (1–4). FAP inhibitors (FAPIs) labeled with radioactive tracers (^68^Ga, ^18^F, or ^177^Lu) are currently utilized in clinical practice for diagnosis and treatment in a wide range of malignant tumors and their associated metastases, demonstrating significant superiority over ^18^F-fluoro-2-deoxy-D-glucose (^18^F-FDG) in certain contexts. FAPI PET/CT has considerable promise for precise cancer assessment (5–9).

Among the extensively studied and reported PET molecular imaging probes, ^68^Ga-FAPI-04 demonstrates a remarkably high tumor-to-background ratio across more than 30 different types of cancer (10–13). However, the application of ^68^Ga-FAPI-04 is limited due to its relatively short half-life (68 min), low overall activity production (only sufficient for 2–3 patients in one batch), and sub-optimal spatial resolution. Due to its longer half-life of 110 min compared to [^68^Ga], [^18^F] facilitates large-scale production and long-distance transportation, making it the most commonly used radioisotope in clinical practice (14, 15). Several ^18^F-labeled FAPIs have been developed for either preclinical or clinical evaluation (16–21). [^18^F]AlF-NOTA-FAPI-04 is one of the ^18^F-labeled FAPIs that has demonstrated superior tumor imaging capabilities in several clinical evaluations, exhibiting improved physical properties, high yields, and favorable imaging characteristics. [^18^F]AIF-NOTA-FAPI-04 has the potential to serve as an ideal radiopharmaceutical for PET imaging (18, 22, 23). However, there are abundant differences in biodistribution between ^18^F-FAPI and ^18^F-FDG. Although ^18^F-FAPI uptake was lower than ^18^F-FDG in most normal tissues, the SUV_mean_ of the gallbladder and pancreas was notably higher in ^18^F-FAPI compared to ^18^F-FDG (24). Previous studies have reported that ^18^F-FAPI demonstrates significant physiological uptake in the gallbladder and biliary tract system, which hampers the detection of their associated malignancies (20, 24). Oral intake of milk after ^18^F-FAPI administration may increase the hepatobiliary clearance rate of ^18^F-FAPI. Full-fat milk can induce the secretion of cholecystokinin(CCK) from the cells of the small intestine mucosa, with effects similar to those observed after direct administration of cholecystokinin, potentially stimulating gallbladder contraction and accelerating the transit of the tracer from the liver to the gastrointestinal tract (25). This approach, which involves the consumption of items such as full-fat milk or milkshakes, is commonly employed in nuclear medicine for myocardial perfusion imaging (26, 27).

The aim of this study was to assess the impact of fat intake on normal abdominal organs uptake of [^18^F] AlF-NOTA-FAPI-04 and to conduct a comparison on the physiological abdominal organ uptake of [^18^F] AlF-NOTA-FAPI-04 and ^18^F-FDG.

## Materials and methods

### Patients

A total of 86 patients who underwent whole-body/abdominal [^18^F]AlF-NOTA-FAPI-04 PET/CT imaging at the Hunan Cancer Hospital between December 2020 and August 2021 were included in our study. Informed consent was obtained from each participant prior to ^18^F-FAPI PET/CT imaging. Patients were divided into two groups according to their diet before the PET scan: treated group, comprised of patients who consumed 250 mL milk 10 ± 5 min after the tracer injection. The volume of the milk was 250 mL, and contained 284 kJ/100 mL, fat content per 100 milliliters was 4.0 g. Control group, permitted no food intake subsequent to the radiotracer administration, which was the standard patient preparation. 64 patients underwent paired ^18^F-FDG and [^18^F]AlF-NOTA-FAPI-04 PET/CT scans.

### Study design

This was a single-center retrospective study conducted at Hunan Cancer Hospital. This research complied with the Declaration of Helsinki’s recommendations for biomedical research involving human subjects and received approval from the Medical Ethics Committee of Hunan Cancer Hospital. Prior to the scan, patients in the treated group did give verbal informed consent to consume milk. The primary endpoint of this study was the physiological ^18^F-FAPI and ^18^F-FDG uptake in the gallbladder, liver, small intestine and pancreas, measured as mean standardized uptake value (SUV_mean_).

### Radiosynthesis and quality control

The F-18 radionuclide was synthesized *in situ* by subjecting O-18-H2O to a 9.8 MeV proton bombardment using a GE MINItrace cyclotron (GE HealthCare, Milwaukee, WI, USA). The FAPI-04 precursor was procured from PET Science and Technology CO., LTD (Beijing, China). [^18^F]AlF-NOTA-FAPI-04 was labeled using the procedure detailed by Jiang et al. (18). The manufacturing of ^18^F-FDG followed the standard procedure, utilizing the coincidence ^18^F-FDG synthesis module (AIO; TRSIS, China). Both [^18^F] AlF-NOTA-FAPI-04 and ^18^F-FDG exhibited a radiochemical purity exceeding 95%. The final product was sterile and met all the requirements stipulated by our institution before to use.

### PET/CT scanning

Patients must strictly fast for 4 h before imaging. The administered intravenous dose of both ^18^F-FAPI and ^18^F-FDG was 3.7 MBq (0.1 mCi)/kg. Fifteen minutes before the ^18^F-FDG injection, height, weight, and fasting blood glucose levels should be measured, with the blood glucose level required to be below 7.0 mmol/L; otherwise, an appropriate amount of insulin should be administered subcutaneously to ensure compliance with the standard. An hour following intravenous delivery, all patients underwent a PET/CT scan on a digital detector scanner (Discovery MI, GE, Healthcare, Milwaukee, WI, USA). The computed tomography (CT) scan covered the area from the whole skull to the upper thighs, using a tube voltage of 110 kV, a tube current of 120 mA, and a slice thickness of 3.75 mm. After the CT scan, a PET scan was done right away in 3D acquisition mode, taking 2 min for each position and 5 to 6-bed positions. Ordered subset expectation maximization (OSEM) was used to construct ^18^F-FDG and ^18^F-FAPI PET/CT images on an Advantage Workstation (AW 4.7, GE HealthCare, Milwaukee, WI, USA). After attenuation correction using the CT data, the reconstructed images were co-registered for analysis. The paired ^18^F-FDG and [^18^F]AlF-NOTA-FAPI-04 PET/CT scans were performed within 14 days.

### ^18^F-FAPI and ^18^F-FDG PET data analysis

All images were independently reviewed by two board-certified nuclear medicine physicians with expertise in interpreting PET/CT examinations. Any discrepancies in the image interpretations were resolved through consensus discussion. The intensity of physiological ^18^F-FAPI and ^18^F-FDG uptake in organs was quantified as the mean standardized uptake value (SUV_mean_). Areas of interest were drawn from tissues on the gallbladder, liver (right lobe), proximal jejunum and pancreas (tail/corpus). The volumes of interest (VOIs) were drawn in three consecutive slices on the PET images focused on the maximum voxel value for the mentioned organs, and the mean values of the SUV in the VOIs were recorded. To minimize a partial volume effect, VOIs were always positioned inside the bounds of the activity distribution. VOIs were delineated at 1 cm for minor tissues and at 2 cm for major organs such as the liver. Additionally, VOIs included intestinal walls and possible luminal content but not extraintestinal content. SUV_mean_ were automatically extracted from the defined VOIs using the AW Workstation.

### Statistical analysis

SPSS (version 25.0; SPSS Inc., Chicago, IL, USA) was employed for the analysis. Continuous variables were expressed as mean ± standard deviation (SD) when the data were normally distributed, otherwise, the median and interquartile range were reported. Categorical variables were represented as percentages (%). Fisher’s exact test or chi-square test was used to compare unordered categorical variables represented as numbers and percentages. Semiquantitative parameters measured using the ^18^F-FAPI and ^18^F-FDG were analyzed using the Mann–Whitney U test, with statistical significance defined by a probability (*p*) value ≤0.05.

## Results

### Participant characteristics

Our cohort initially enrolled 105 consecutive patients, however, after excluding 15 patients with cholecystectomy and 4 patients with poor image quality, a total of 86 patients were ultimately included for the evaluation of the effect of pre-scan dietary preparations on the physiologic ^18^F-FAPI and ^18^F-FDG uptake of the gallbladder. The characteristics of the patients are summarized in Table 1. The treated group comprised 67 patients who drank milk subsequent to the radiotracer administration, while the Control group included 19 patients who underwent no food intake after the tracer injection. No statistically significant differences were observed between the two groups of patients in terms of age, gender, weight, body mass index, injection dose, and history of gastrectomy. All patients tolerated this test well, with no drug-related pharmacologic effects or physiologic reactions. No patient noticed any symptoms or experienced any adverse reactions during the injection process until the end of the examination.

**Table 1:** Patients’ characteristics of the treated group and control group (*n* = 86).

Characteristics	Treated (*n* = 67)	Control (*n* = 19)	*p*-value
Age, years	54.34 ± 11.63	49.0 ± 10.82	0.076
Gender			
Male	36	9	0.624
Female	31	10	
Weight, kg	56.0 ± 11.62	57.42 ± 9.59	0.627
Height, cm	160.57 ± 7.56	160.05 ± 7.91	0.796
BMI, kg/m2	21.61 ± 3.59	22.47 ± 3.71	0.361
Injected dose, MBq	236.3 ± 34.45	242.3 ± 24.85	0.477
Resection			
Gastric resection	18	5	0.962
Non-gastric resection	49	14	

### Comparison of physiological ^18^F-FAPI uptake in treated group versus control group

**Figure 1:**
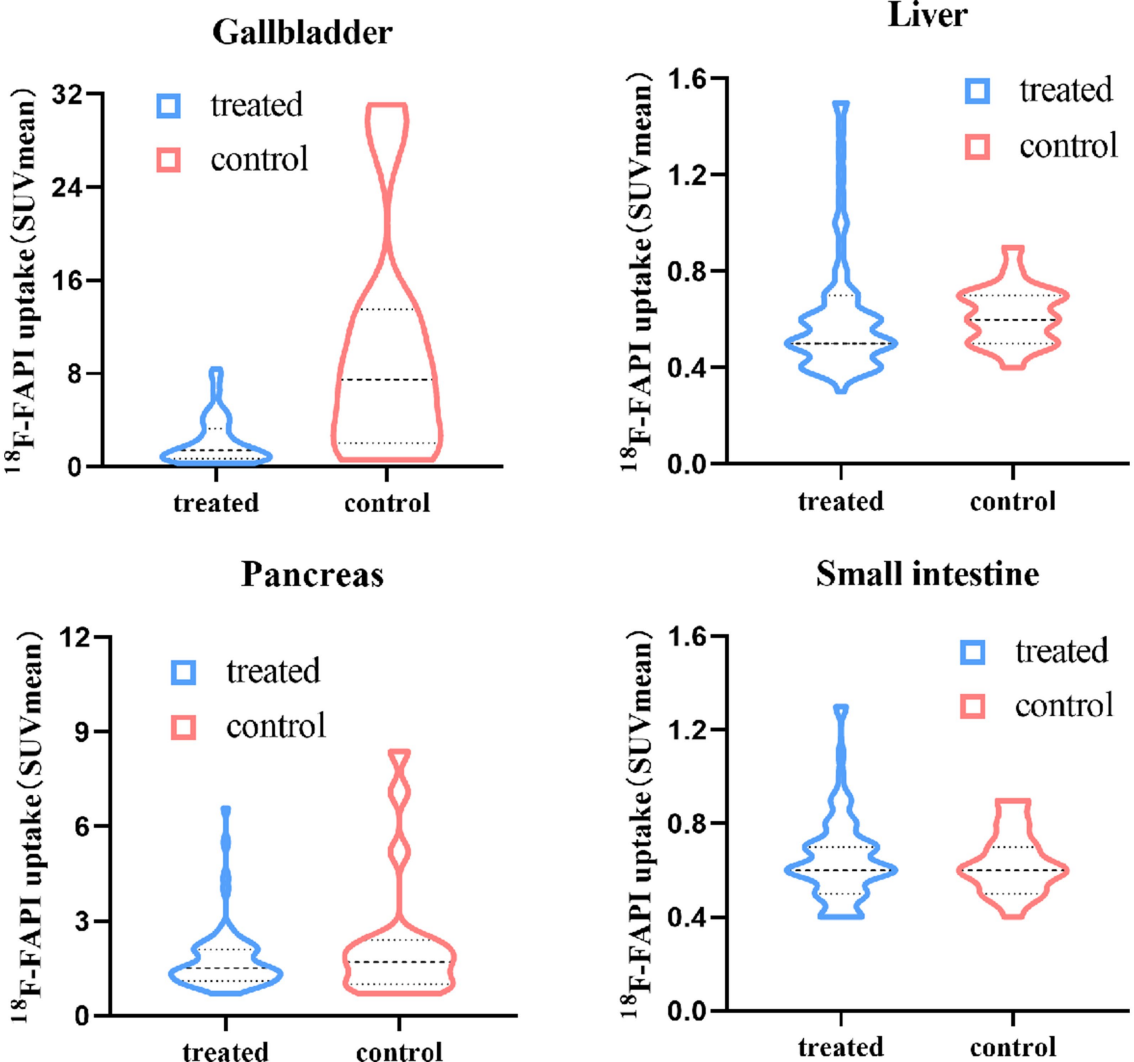
Physiological ^18^F-FAPI uptake in gallbladder, liver, small intestine and pancreas for different food intake protocols.

The physiological ^18^F-FAPI uptake in various organs for the two groups are presented in Table 2. Quantitative analysis revealed moderate-to-low uptake in the average SUV_mean_ in the liver, small intestine and pancreas. No significant differences were observed in the physiologic ^18^F-FAPI uptake in these organs between treated group and control group. There was a statistically significant difference in the ^18^F-FAPI uptake in the gallbladder between the treated group and the control group (*p* < 0.001). The average SUV_mean_ in the treated group was 2.19 ± 2.01, which was significantly lower than the average SUV_mean_ of 10.04 ± 9.66 in the control group. Figure 1 illustrates the distribution of physiological tracer uptake in the gallbladder, liver, small intestine and pancreas between the treated group and control group.

**Table 2:** Physiological ^18^F-FAPI uptake (SUV_mean_) per food intake protocol and per organ.

Organ	Treated (*n* = 67)	Control (*n* = 19)	*p*-value
Gallbladder	2.19 ± 2.01	10.04 ± 9.66	<0.001
Liver	0.80 ± 1.08	0.62 ± 0.12	0.216
Small intestine	0.65 ± 0.19	0.64 ± 0.14	0.923
Pancreas	1.78 ± 1.02	2.92 ± 3.60	0.545

Values are presented as average and standard deviation.

### Comparison of physiological ^18^F-FAPI uptake and physiological ^18^F-FDG uptake in treated group/control group

Subgroup analysis was performed on patients (*n* = 64) who underwent both ^18^F-FAPI and ^18^F-FDG PET/CT scan, In the treated group and control group, the ^18^F -FAPI uptake of the liver (*p* ≤ 0.001) and small intestine (*p* ≤ 0.001) were significantly lower compared to ^18^F-FDG uptake. However, in the treated group and control group, the ^18^F-FAPI uptake of the gallbladder (*p* ≤ 0.002) and pancreas (*p* ≤ 0.005) were significantly higher compared to ^18^F-FDG uptake (Table 3).

**Table 3:** Physiological ^18^F-FAPI and ^18^F-FDG uptake (SUV_mean_) in treated group and control group.

Organ	Treated (*n* = 51)			Control (*n* = 13)		
	^18^F-FAPI	^18^F-FDG	*p*-value	^18^F-FAPI	^18^F-FDG	*p*-value
Gallbladder	2.41 ± 2.18	0.76 ± 0.39	<0.001	9.30 ± 9.63	0.83 ± 0.35	0.002
Liver	0.87 ± 1.23	2.10 ± 0.68	<0.001	0.62 ± 0.14	1.85 ± 0.30	0.001
Small intestine	0.68 ± 0.20	1.45 ± 0.47	<0.001	0.68 ± 0.13	1.18 ± 0.27	0.001
Pancreas	1.90 ± 1.12	1.37 ± 0.47	0.002	3.33 ± 3.09	1.26 ± 0.17	0.005

### Comparison of physiological ^18^F-FAPI uptake (SUV_mean_) in treated group/control group after gastric resection

**Figure 2:**
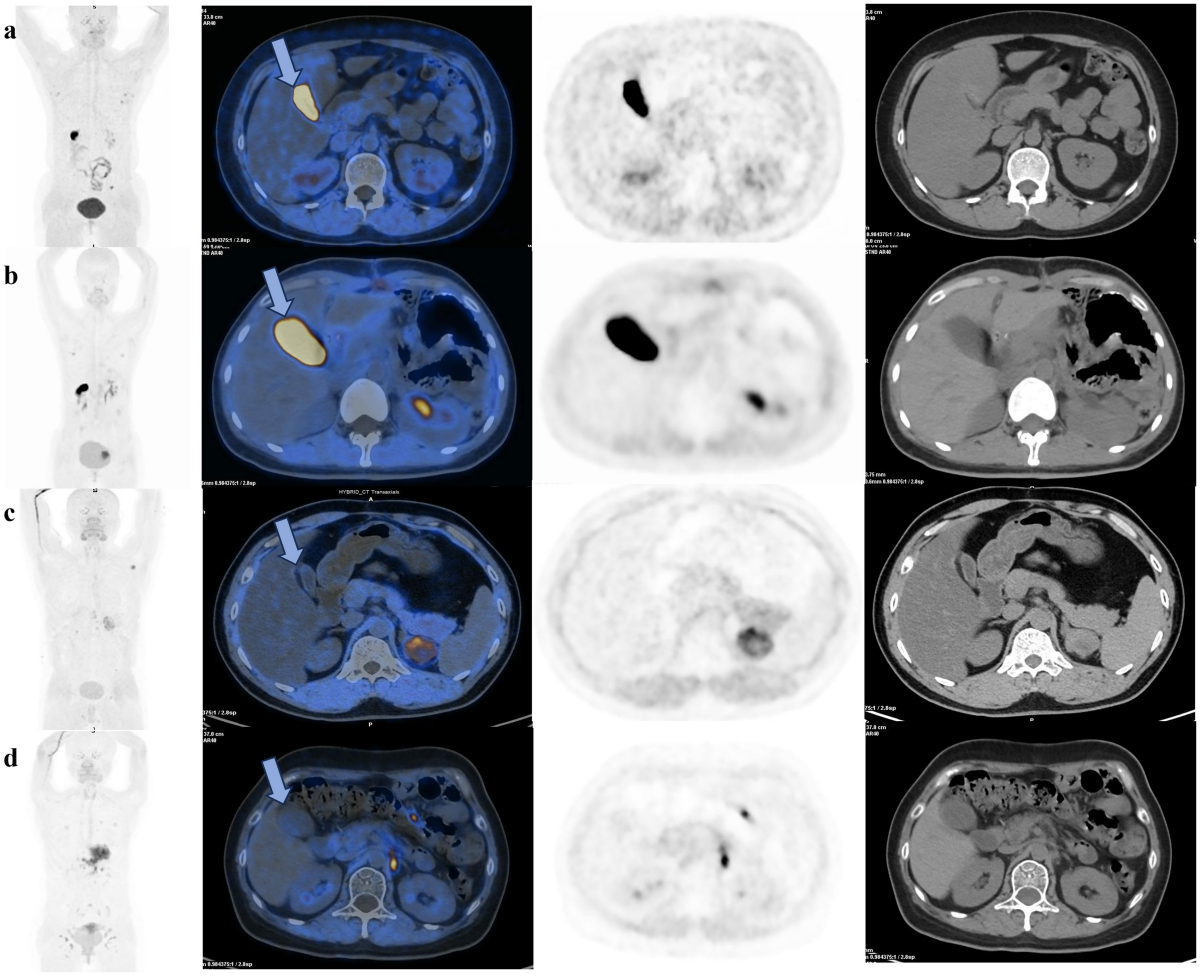
^18^F-FAPI PET/CT scans of cancer patients demonstrate that pre-scan milk has a significant effect on reducing physiological uptake in the gallbladder after gastric resection or without gastric resection. (a) A patient without gastric resection in the control group showed significantly ^18^F-FAPI uptake in the gallbladder (blue arrow). (b) A patient after gastric resection in control group, with increased physiological uptake in the gallbladder (blue arrow). (c) A patient without gastric resection in the treated group, without visible gallbladder uptake (blue arrow). (d) A patient after gastric resection in the treated group, without visible gallbladder uptake (blue arrow) due to gallbladder emptying.

In a subgroup analysis of gastrectomy patients, there was significant difference in physiologic gallbladder uptake between two groups(*p* = 0.04), the average SUV_mean_ in the treated group was 2.56 ± 2.12, which was significantly lower than the average SUV_mean_ of 14.62 ± 14.71 in the control group. Apart from this, there were no difference in the physiologic ^18^F-FAPI uptake of liver, small intestine and pancreas between treated and control group after gastric resection (Table 4). Figure 2 illustrates the clear visual difference in the physiological ^18^F-FAPI uptake of gallbladder between the treated group and control group after gastric resection or without gastric resection.

**Table 4:** Physiological ^18^F-FAPI uptake (SUV_mean_) in treated group and control group after gastric resection.

Organs	Treated (*n* = 18)	Control (*n* = 5)	*p*-value
Gallbladder	2.56 ± 2.12	14.62 ± 14.71	0.04
Liver	0.64 ± 0.29	0.64 ± 0.05	0.234
Small intestine	0.81 ± 0.23	0.74 ± 0.13	0.596
Pancreas	2.37 ± 1.39	4.30 ± 3.20	0.191

## Discussion

Radiolabelled FAPI has been reported to achieve better results in a variety of tumor imaging and is considered a suitable alternative to ^18^F-FDG (28, 29). The application of ^68^Ga-FAPI-04 is restricted due to its relatively short half-life, low overall activity production, and sub-optimal spatial resolution. On the other hand, ^18^F-labeled FAPIs have shown to possess superior tumor imaging abilities in various clinical evaluations, which exhibit improved physical properties, high yields, and favorable imaging characteristics (14, 15). Nevertheless, previous research demonstrated that ^18^F-FAPI has a generally high physiologic uptake in the normal gallbladder, reducing the diagnostic accuracy of primary and metastatic gallbladder lesions (20, 22, 24). We assessed the impact of pre-PET/CT ingestion of milk on the biodistribution of ^18^F-FAPI within normal abdominal organs in a tumor patient cohort.

In this study, physiological uptake of ^18^F-FAPI in the gallbladder was significantly lower in the treated group patients compared to the control group, which facilitates the visualization of gallbladder tumors. The principle of decreased gallbladder uptake of ^18^F-FAPI is based on the physiological metabolic characteristics of ^18^F-FAPI. Previous experiments on animals have indicated that ^18^F-FAPI is mainly excreted through the urinary and biliary systems (16). FAPI is a lipophilic tracer that can be excreted into the intestine by binding to bile acids in the biliary system (16, 18, 30). According to Heraghty’s study (31), a fatty meal can increase the hepatobiliary clearance of contrast agent, which is consistent with our findings. It is noteworthy that among the control group, two patients with the highest gallbladder uptake had undergone gastric cancer surgery years ago. Postoperative metabolic changes may alter bile acid production and lead to the formation of biliary sludge, increasing the incidence of gallbladder pathology and thus affecting gallbladder uptake (32).

Furthermore, we compared the results of 64 patients who underwent both ^18^F-FDG and ^18^F-FAPI PET/CT scans. We observed the physiologic ^18^F-FAPI uptake by the liver and small intestine in the treated group and control group was lower than ^18^F-FDG, and the ^18^F-FDG uptake of gallbladder and pancreas was significantly lower than the ^18^F-FAPI uptake in the treated group and control group, which is aligned with the previous studies (20, 22, 24, 33). Our study demonstrated that ^18^F-FAPI PET/CT can effectively reduce the physiological uptake of liver and small intestine, improving the ^18^F-FAPI visualization, thereby improving the lesion detection rate. A higher background ^18^F-FAPI uptake in gallbladder and pancreas might unbeneficial in detecting tumors and metastatic lesions in the abdominal cavity. However, one study has indicated that FAPI-PET is a reliable diagnostic method for pancreatic cancers (34), which suggests that a minor difference of SUV_mean_ between ^18^F-FAPI and ^18^F-FDG cannot affect the accuracy of diagnosis in this type of cancer. We hypothesized that the slight increase in gallbladder uptake of treated group on ^18^F-FAPI PET/CT did not affect the detection of gallbladder lesions.

In the subgroup analysis of gastrectomy patient, the treated group and control groups did not exhibit substantial changes in liver, small intestines and pancreas uptake. However, the physiologic ^18^F-FAPI uptake of gallbladder in the treated group was significantly lower than the control group. There is a lack of definitive studies on physiologic ^18^F-FAPI uptake in gastrectomy patient. The physiological mechanisms related to the effect of fat intake on gallbladder contraction after gastrectomy remain unclear. Inoue. K highlighted that the release of CCK serves as the chief mechanism through which the ingestion of a fatty meal causes contraction of the gallbladder even after gastrectomy as well as before gastrectomy (35). Watanapa. P suggested that hyper-cholecystokininaemia persists for up to 15 months and may even increase with time after gastrectomy (36). However, some studies showed delayed emptying of the gallbladder after a gastric resection or vagotomy. The contraction of the gallbladder is caused by the stimulation of the vagal nerve and impaired gallbladder motor function could result from vagal denervation (37). Our study shows that milk consumption similarly promotes gallbladder emptying and decreases physiological gallbladder ^18^F-FAPI uptake in gastrectomy patients, which supports a major role for CCK in gallbladder contraction after gastrectomy.

Our study has several limitations: firstly, the sample size was small and the number of patients in the two groups were unbalanced. Statistical analysis may lack generalizability, and the conclusion need to be verified in larger studies. Secondly, SUV_bw_ (normalized by Body Weight) is occasionally overestimated, particularly in obese individuals, which can lead to systematic bias for serial scans of patients with multiple follow-ups throughout the course of treatment. Our study would benefit from SUV measures normalized by lean body mass (38). Thirdly, the ^18^F-FAPI uptake of gallbladder was higher than the ^18^F-FDG uptake in the treated group. Consideration of ^18^F-FAPI or ^18^F-FDG for visualization is crucial in the comprehensive assessment of gallbladder cancer patients. Despites these limitations, it is believed that this study has undoubtedly enhanced our understanding of the influence of fat intake on [^18^F] AlF-NOTAFAPI-04 uptake in the normal abdominal organs.

## Conclusion

In this retrospective study, we showed that the ^18^F-FAPI uptake of gallbladder in treated group was significantly lower than control group, which suggested that consumption of 250 mL of milk after the tracer injection potentially stimulate gallbladder contraction. Integration of pre-scan milk into routine ^18^F-FAPI PET/CT may enhance identification of gallbladder lesions, and may improve the diagnosis of gallbladder cancer in the future. On the other hand, the ^18^F-FAPI uptake of liver and small intestine was significantly lower than the ^18^F-FDG uptake, and the ^18^F-FDG uptake of gallbladder and pancreas was significantly lower than the ^18^F-FAPI uptake in both the treated group and the control group. ^18^F-FDG and ^18^F-FAPI serve as complementary tracers, thus dual tracer imaging holds significant clinical value.

## Data Availability

The raw data supporting the conclusions of this article will be made available by the authors, without undue reservation.

## Supplementary material

SUPPLEMENTARY FIGURE S1 Schematic diagram of the volume of interest (VOI) delineation. The volumes of interest (VOIs) were drawn in three consecutive slices on the PET images focused on the maximum voxel value for the mentioned organs, and the mean values of the SUV in the VOIs were recorded. VOIs were delineated at 1 cm for minor tissues and at 2 cm for major organs such as the liver. The VOI of gallbladder and small intestine are located within the lumen, excluding the wall.
