# Supplementary figures and images for: Impact of fat intake on [18F]AlF-NOTA-FAPI-04 uptake in normal abdominal organs
Source: Front Med (Lausanne). 2024 Nov 7;11:1464779. doi: 10.3389/fmed.2024.1464779 (PMC11578823; doi:10.3389/fmed.2024.1464779)

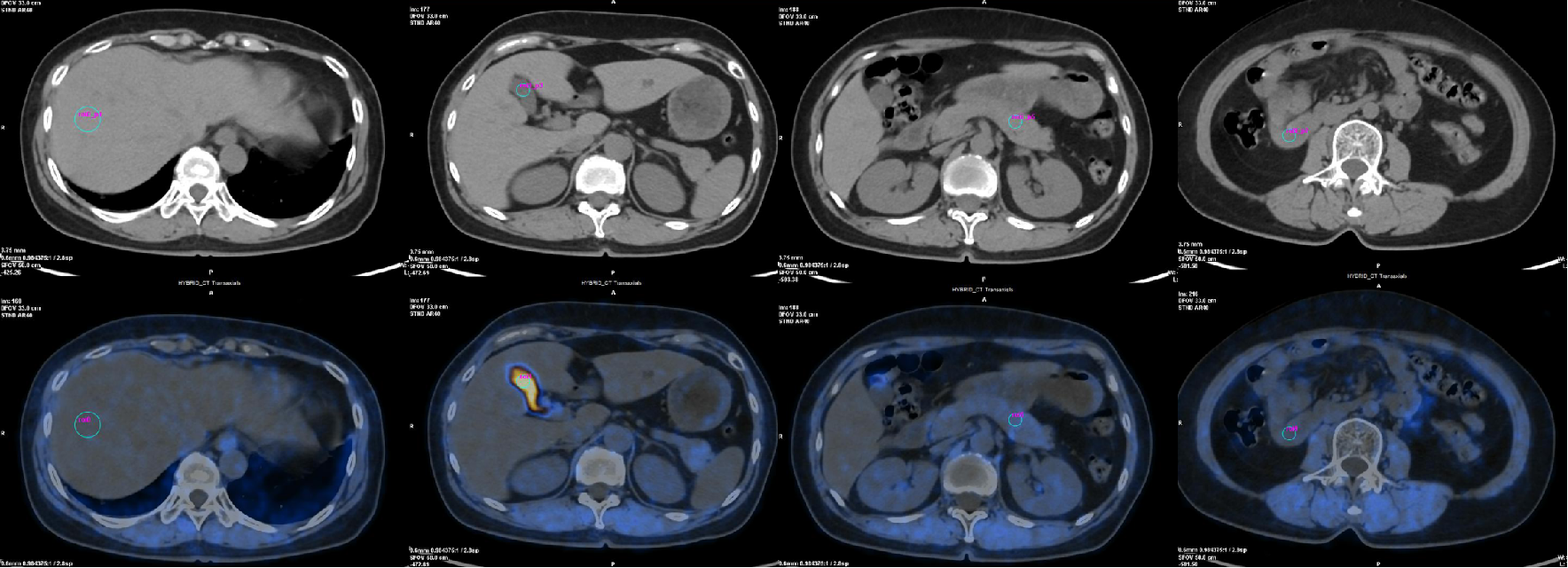

Supplement: SUPPLEMENTARY FIGURE S1 — Schematic diagram of the volume of interest (VOI) delineation. The volumes of interest (VOIs) were drawn in three consecutive slices on the PET images focused on the maximum voxel value for the mentioned organs, and the mean values of the SUV in the VOIs were recorded. VOIs were delineated at 1 cm for minor tissues and at 2 cm for major organs such as the liver. The VOI of gallbladder and small intestine are located within the lumen, excluding the wall. [file Image_1.TIF]
